# Exogenous glucocorticoid dose impacts circulating microRNA expression in patients with adrenal insufficiency due to 21-hydroxylase deficiency

**DOI:** 10.3389/fendo.2026.1784619

**Published:** 2026-03-31

**Authors:** Vipula Kolli, Jacob Trick, Annie Schulman, James R Iben, Cameron Padilla, Tianwei Li, Fabio Rueda Faucz, Deborah P. Merke

**Affiliations:** 1 Department of Pediatrics, National Institutes of Health Clinical Center, Bethesda, MD, United States; 2 The Eunice Kennedy Shriver National Institute of Child Health and Human Development, Bethesda, MD, United States; 3 Hospital for Endocrine Surgery Research Center, HCA Florida Healthcare, HCA Healthcare Research Institute, Tampa, FL, United States

**Keywords:** 21-hydroxylase deficiency, adrenal insufficiency, congenital adrenal hyperplasia, enrichment analysis, glucocorticoid, microRNA

## Abstract

**Introduction:**

Patients with adrenal insufficiency due to 21-hydroxylase deficiency (21-OHD) are often exposed to supraphysiologic doses of glucocorticoids (GCs), which can augment metabolic dysfunction by altering glucose, lipid and protein metabolism. Exogenous GCs also fail to mimic the natural circadian rhythm of cortisol secretion. MicroRNAs (miRNAs) are important regulators of gene expression involved in various biological and pathological processes. GCs can regulate miRNAs and in turn miRNAs can modulate GC receptor (GR) activity influencing GR-mediated activity. In this pilot study, we examine the effects of exogenous GCs on circulating miRNA levels in patients with adrenal insufficiency due to classic 21-OHD.

**Patients/methods:**

Blood samples were collected from 37 patients with classic 21-OHD. Comprehensive miRNA-seq transcriptomic profiling was performed followed by functional enrichment analysis. Patients on different GC types (short- *vs.* long-acting) and on different GC doses (physiologic, moderate, or high dose;<12 mg/m^2^/day, 12-18 mg/m^2^/day, >18 mg/m^2^/day hydrocortisone equivalents (HCe), respectively) were compared.

**Results:**

Seven miRNAs (hsa-miR-320a, hsa-miR-122, hsa-let-7b, hsa-let-7i, hsa-miR-4747, hsa-miR3591, hsa-miR-4732) were differentially expressed in patients receiving high-dose GC therapy compared to those receiving physiologic doses (*P* < 0.05). Expression of all 7 miRNAs was negatively correlated with GC dose (*P* ≤ 0.03). Gene ontology enrichment and KEGG pathway analyses of sequencing results found that the differentially expressed miRNAs were involved in cell cycle progression and proliferation, insulin signaling, metabolic dysfunction, circadian rhythm, and GR signaling. Furthermore, MIENTURNET integrative pathway and network analyses of miRNAs identified 470 target genes, many of which play a role in insulin sensitivity, lipid metabolism, gluconeogenesis, oxidative stress and/or tumor metabolism. Conversely, no differences were observed in miRNA profiles between patients who were on short-acting compared to long-acting GCs.

**Conclusions:**

In patients with CAH, daily GC dose > 18 mg/m^2^/day HCe impacts the expression of miRNAs that are known to be associated with GCs and their biological effects. The identification of a distinct circulating miRNA signature associated with supraphysiologic GC exposure in patients with CAH suggests that miRNAs could emerge as a valuable non-invasive biomarker for detecting GC excess and monitoring treatment.

## Introduction

Classic congenital adrenal hyperplasia (CAH) due to 21-hydroxylase deficiency (21-OHD) is the most common cause of adrenal insufficiency during childhood, and overall the most common genetic cause of primary adrenal insufficiency (1). It is an autosomal recessive disorder of steroidogenesis resulting in a characteristic pattern of hormone imbalances including glucocorticoid (GC), mineralocorticoid, and epinephrine deficiencies, and androgen excess (2). The decreased production of cortisol interferes with the negative feedback inhibition of the hypothalamic-pituitary-adrenal (HPA) axis resulting in an overproduction of pituitary ACTH and, along with cortisol precursor buildup, an overproduction of adrenal androgens. The combination of cortisol, aldosterone, and epinephrine deficiencies places patients at risk for life-threatening adrenal crises, during which patients may experience acute episodes of hyperkalemia, metabolic acidosis, and hypoglycemia (2, 3). Other adverse clinical outcomes of CAH include altered growth and early puberty, cardiovascular disease risk, decreased bone mineral density, and compromised reproductive health (4, 5).

The treatment of CAH aims to replace deficient hormones, prevent and treat adrenal crises, and suppress excess adrenal androgens. Adverse outcomes are due to the combination of disease-related and treatment-related factors (2). Supraphysiologic doses of GC are often needed to counter the ACTH-driven excess androgen production by the adrenal glands (2). In addition to replacing cortisol, GCs play an important role in maintaining and regulating various functions like metabolism, immune response, cardiovascular function, mood, and stress response (6). Prolonged exposure to excess GC can be deleterious to the body causing metabolic syndrome, cardiovascular risk, weight gain, decreased bone mineral density, mood disorders, and increased risk of infections. GCs can increase metabolic dysfunction by affecting glucose, lipid, and protein metabolism (7). Moreover, standard GC treatment regimens fail to effectively mimic the physiologic circadian rhythm (8). In healthy individuals, cortisol peaks in the morning upon awakening and is low in the evening, which is synchronous with the active phase of life and exhibits a strong circadian rhythm. This daily rhythm of circulating GCs is a strong modulator of various physiological processes (9, 10).

MicroRNAs (miRNAs) are noncoding, single-stranded RNA segments of 18-24 nucleotides in length, and they play a pivotal role in post-transcriptional regulation of gene expression (11). MiRNAs play an essential role in various biological processes in normal development like proliferation, differentiation, and apoptosis. Aberrant changes in miRNA expression are associated with disease conditions (11). For example, dysregulation in specific miRNAs can lead to changes in cell proliferation, tumor progression (12), and metabolic syndrome where lipid and glucose homeostasis are affected (13). Studies have also shown that miRNAs can act as tumor suppressors or oncogenes (12). The majority of miRNAs are released into extracellular fluids like plasma, serum, urine, saliva, semen, and cerebrospinal fluid (14). Within the endocrine system, miRNAs have been shown to modulate the expression of enzymes involved in steroidogenesis mainly by altering the availability of GCs and their receptors, and by modulating receptor activity and signaling (15). Furthermore, GC production has been shown to impact miRNA expression (7, 16). This complex reciprocal relationship between GCs and miRNA suggests that hormone-miRNA feedback loops may play an important role in endocrine regulation and provides a conceptual framework for differential miRNA expression in endocrine disease (17–19). Circulating miRNAs are dynamic and have also been studied as potential biomarkers for adrenal diseases (14).

To fully understand the unique pathophysiology of CAH, it is essential to define the impact of GCs on health and disease. This is the first study to evaluate the role of circulating miRNA in CAH. This pilot study provides comprehensive miRNA profiling of CAH patients who are receiving GC treatment, thus providing new insights into the GC-specific effects on patient outcomes.

## Patients and methods

### Patients

Patients with classic CAH due to 21-OHD were enrolled in the Natural History study at the National Institutes of Health Clinical Center (NCT#00250159). All studies were approved by the National Institute of Health Institutional Review Board. Diagnosis was confirmed by hormonal testing and *CYP21A2* genotyping, as previously described (20). All adult patients provided informed consent. Blood samples were collected in PAXgene Blood RNA tubes (BD Life Sciences, Franklin Lakes, MD) from 37 patients in the early morning around 8AM before GCs were administered. Tubes were allowed to sit at room temperature for 2-3 hours and subsequently stored at -80 °C until used. GCs were categorized as either short-acting (hydrocortisone) or synthetic long-acting (prednisone, methylprednisolone, or dexamethasone) based on half-life. GC dosages were evaluated as hydrocortisone equivalents (HCe) (mg/m^2^/day; hydrocortisone: mg x1, prednisone/prednisolone: mg x5, methylprednisolone: mg x6, dexamethasone: mg x80) (21–23). GC doses were categorized as physiologic (<12 mg/m^2^/day), moderate dose (12-18 mg/m^2^/day), and high dose (>18 mg/m^2^/day). The definition of physiologic GC dose was based on studies of normal cortisol production rates in healthy individuals (24, 25) and has been used in clinical trials aiming to reduce daily GC dose to physiologic ranges in patients with classic CAH (26–28). Although GC doses >18 mg/m^2^/day are not recommended and have been shown to stunt growth in children (2, 29–31), they are sometimes used clinically to control ACTH-driven hyperandrogenism and poor disease control.

### miRNA isolation and sequencing

PAXgene Blood RNA tubes containing whole blood were centrifuged at 3000-5000g for 10 min to separate out the nucleic acids pellet. The RNA pellet was resuspended in RNase-free water, before being centrifuged at 3000-5000g for 10 minutes a second time. Total miRNA was extracted using the Qiagen PAXgene Blood miRNA Kit for microRNA isolation (QIAGEN, Germantown, MD) according to the manufacturer’s instructions. RNA yield, quality, and size were assessed through RNA 6000 Nano Assays using a 2100 Agilent Bioanalyzer (Agilent Technologies, Santa Clara, CA). Sequencing libraries were constructed using QIAseq miRNA Library Kit with QIAgen miRNA 96 Index IL (QIAGEN, Valencia, CA) according to the manufacturer’s protocol. Libraries were indexed and sequenced using Novaseq 6000 (Ilumina, San Diego, CA). Sequencing data was trimmed with cut adapt (-a AACTGTAGGCACCATCAAT -a AGATCGGAAGAGCACACGTCTGAACTCCAGTCA --overlap 6 -q 20 --minimum-length 18), aligned using STAR aligner with the following miRNA-specific parameters (--outFilterMismatchNmax 1 --outFilterMultimapScoreRange 0 --outFilterMatchNmin 16 --alignSJDBoverhangMin 1000 --alignIntronMax 1 --outFilterMatchNminOverLread 0 --outFilterScoreMinOverLread 0). MiRNA-sequencing data were aligned to the reference human GRCh38 genome assembly using RNA-STAR v2.7.3a against GENCODE human GRCh38, and miRNA was quantitated with ENSEMBL gene index definitions with subread featureCounts v1.6.4. Differential expression testing between various treatment sets was performed with DESeq2 (32).

### Functional enrichment analysis

The Gene Ontology (GO) of biological functions and Kyoto Encyclopedia of Genes and Genomes (KEGG) pathway enrichment analyses were performed on the differentially expressed miRNAs using DIANA-miRPath v4.0 (33). The GO analysis included the Biological Process (BP) terms to provide insight into the biological systems affected by the differentially expressed miRNA. Enrichment bar graphs were created for GO biological processes and KEGG pathways for the *Homo sapiens* using ggplot2.

### miRNA-mRNA network construction

MicroRNA Enrichment TURned NETwork (MIENTURNET) (34), a web-based tool, was used to create a network of prioritized target genes of the differentially expressed miRNAs co-expressed in the high *vs* physiologic dose GC analysis. MiRNA-target gene interactions were identified using miRTarBase v.7.0. A hypergeometric test was used to determine if each target gene was enriched in the miRNA-target database. The resulting *P*-values were corrected for multiple hypotheses to provide a False-Discovery Rate (FDR) associated with each target gene. The miRNA-mRNA network was visualized using ggraph in R.

### Statistical analysis

Wald testing for differential expressions of miRNA across conditions was performed using a negative binomial generalized linear model (NB GLM) using DESeq2 lfcShrink with adaptive shrinkage (ASHR) to reduce high-variance noise. *P*-values were adjusted for multiple testing using Benjamini-Hochberg (BH) correction. In the identification of differentially expressed miRNAs and functional enrichment analyses, an FDR *q* < 0.05 was used as cutoff for significance. Linear regression analyses were performed for each differentially expressed miRNA in R (version 4.5.2) using ordinary least squares. For each analysis, miRNA expression values were modeled as a function of GC dose using the lm() function in base R. Fisher’s exact test was used to evaluate GC type (short-acting vs. long-acting) across the different GC dose categories.

## Results

### Patients

Thirty-seven patients (20 males, 17 females; median age 28 (IQR of 22.0-34.0) with classic 21-OHD participated (**Table 1**). About half were receiving short-acting hydrocortisone and half were receiving long-acting GC with median HCe of 15.2 [11.7-20.2] mg/m^2^/day at the clinic visit when miRNA was obtained; 43% had a normal BMI, and 27% were receiving daily doses of GC in the physiologic range (< 12 mg/m^2^/day) (**Table 1**). Patients receiving synthetic long-acting GCs were more likely to be receiving higher GC dose (*P =* 0.007).

**Table 1:** Clinical characteristics of patients with congenital adrenal hyperplasia.

Characteristics	Physiologic (n = 10)	Moderate (n = 14)	High (n = 13)
Age (years)	33.5 (30.8-36.8)	27.5 (22.3-34.0)	25.0 (20.0-29.0)
Female	6 (60.0%)	4 (28.6%)	7 (53.8%)
Phenotype			
Salt-wasting	6 (60.0%)	10 (71.4%)	9 (69.2%)
Simple-virilizing	4 (40.0%)	4 (28.6%)	4 (30.8%)
Glucocorticoid Type			
Short-Acting	8 (80.0%)	6 (42.9%)	2 (15.4%)
Long-Acting	2 (20.0%)	8 (57.1%)	11 (84.6%)
Hydrocortisone Equivalence^1^ (mg/m^2^/day)			
At Visit	10.3 (8.6-11.2)	15.0 (13.6-15.6)	23.8 (20.4-26.8)
Over prior 60 months	10.7 (10.2-11.4)	15.8 (14.1-17.3)	21.2 (18.8-26.3)
BMI Classification			
Normal (<25 kg/m^2^)	4 (40.0%)	7 (50.0%)	5 (38.5%)
Overweight (25-30 kg/m^2^)	4 (40.0%)	4 (28.6%)	6 (46.2%)
Obese (> 30 kg/m^2^)	2 (20.0%)	3 (21.4%)	2 (15.4%)
Androstenedione^2^			
Suppressed	2 (20.0%)	1 (7.1%)	2 (15.4%)
Optimal	6 (60.0%)	7 (50.0%)	6 (46.2%)
Elevated	2 (20.0%)	6 (42.9%)	5 (38.5%)
17-OHP (ng/dL)	1646 (442-3068)	2245 (352-10807)	505 (241-9298)
Insulin resistance^3^	3 (33.3%)^4^	7 (50.0%)	6 (54.5%)^5^
Low bone mineral density^6^	1 (14.3%)^7^	3 (21.4%)	3 (25.0%)^8^

Data presented as n (%) or median (interquartile range). Adjusted for missing data.

^1^Hydrocortisone dose equivalent: prednisone: mg x5, methylprednisolone: mg x6, dexamethasone: mg x80. ^2^Based on age- and sex-specific normal range.^3^ Defined as HOMA-IR above 2.5.^4^n=9 due to missing laboratory values. ^5^n=11 due to missing laboratory values. ^6^Defined as Z-score ≤ −2 at the total hip, femoral neck, or AP spine. ^7^n=7 due to missing laboratory values. ^8^n=12 Due to missing laboratory values.

Sex differences in GC dose were not observed.

### Glucocorticoid impact on miRNA expression

**Figure 1:**
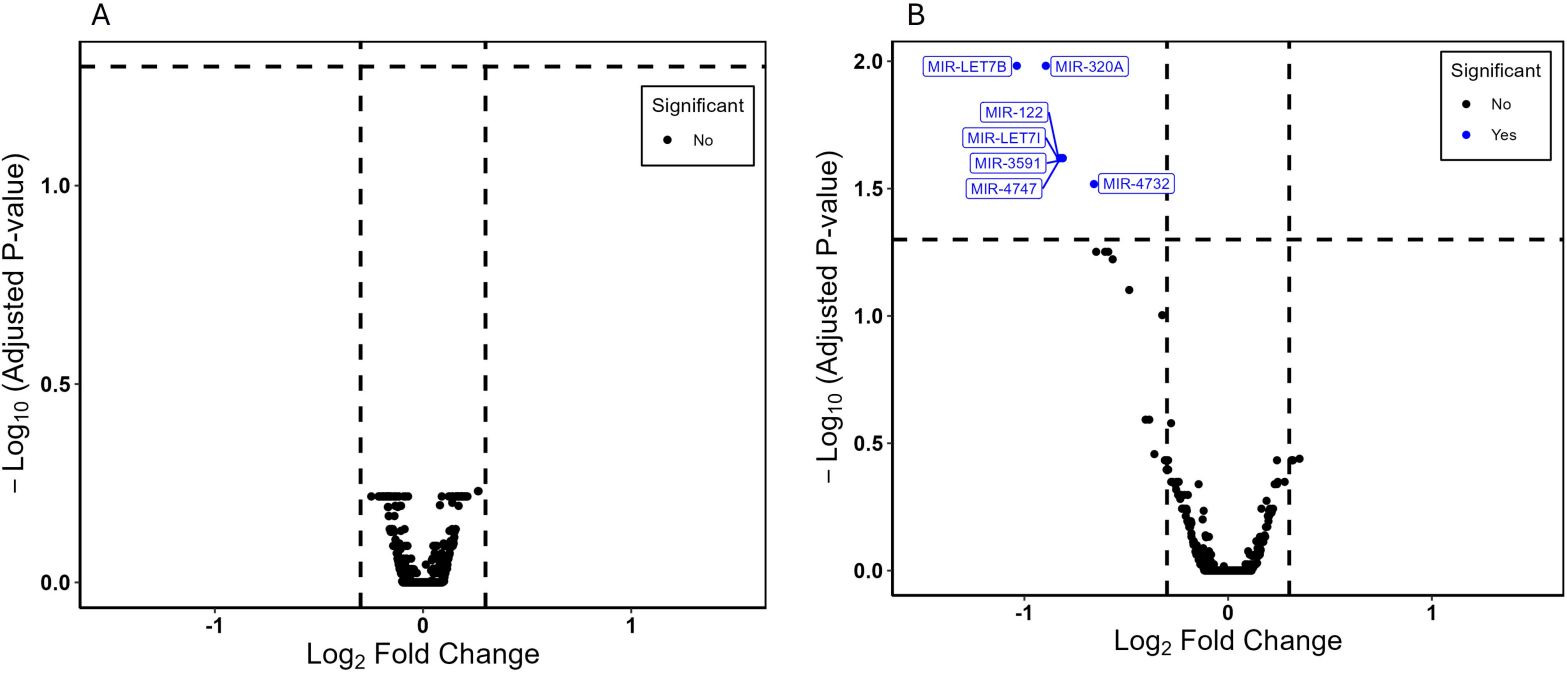
Differentially expressed miRNAs. Volcano plots showing the relationship between fold change (Log2 fold change on X-axis) and statistical significance (-Log10 Adjusted *P*-value on Y-axis). **(A)** Patients receiving short-acting (n=16) and long-acting (n=21) glucocorticoids. No differences in miRNA expression were observed between the GC types. **(B)** Patients receiving physiologic (n=10;<12 mg/m^2^/day) and high (n=13; >18 mg/m^2^/day) glucocorticoid dose, measured in hydrocortisone equivalents. Blue points represent the seven differentially expressed miRNAs with false discovery rate *q<* 0.05, considered statistically significant.

**Figure 2:**
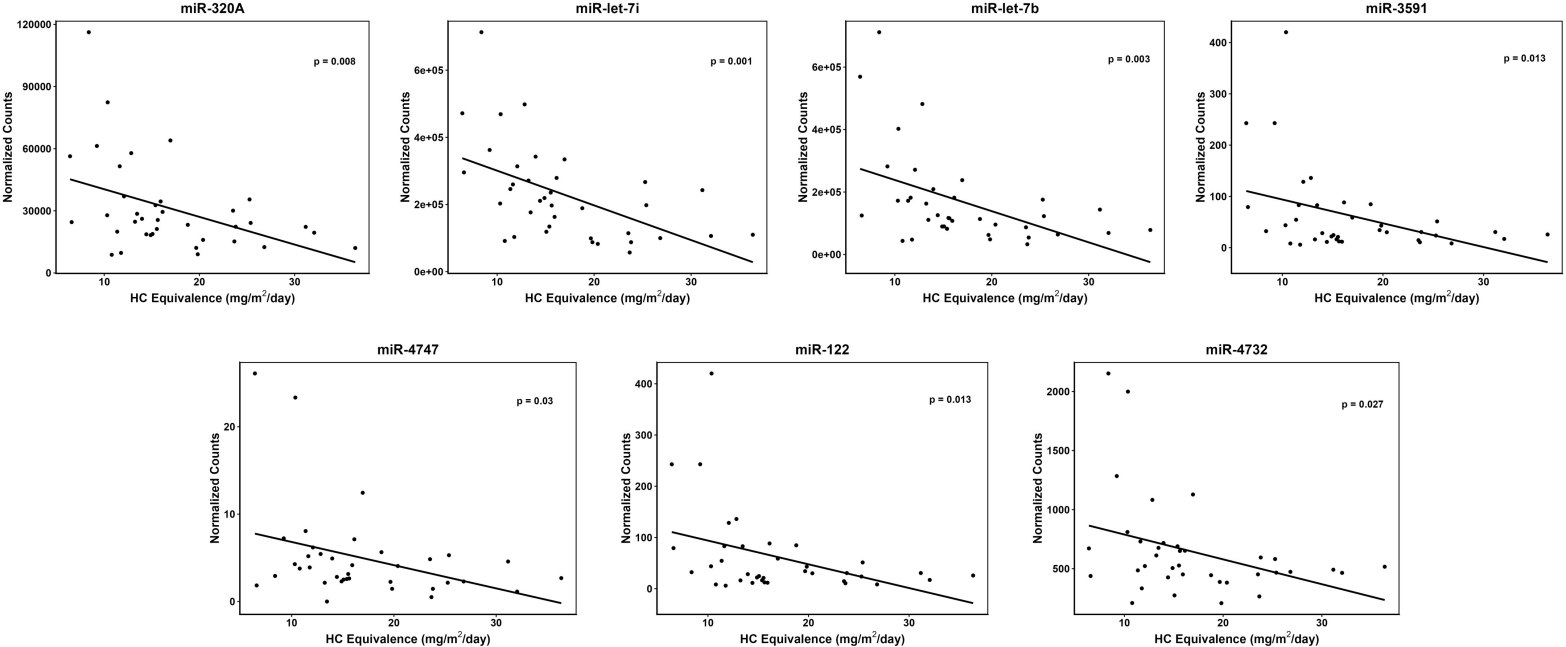
Differentially expressed miRNAs between patients on different glucocorticoid doses. Shown is the negative correlation between miRNA expression levels measured by normalized counts (Y-axis) and glucocorticoid dose measured in hydrocortisone equivalents (X-axis).

Overall, 1198 of 1879 annotated miRNAs (GENCODE human v43) were detected across samples which were used in differential expression testing, all samples were used in the global model for miRNA gene normalization and dispersion estimation. To determine whether GC duration of action and potency might play a role in miRNA expression, miRNA signatures were compared between patients receiving short-acting (n=16) hydrocortisone and synthetic long-acting GCs (n=21): no differences were observed (adjusted *P*-value< 0.05, $|log_{2}\left(FC\right)|>\;0.3$) between the two groups (**Figure 1A**). However, differential miRNA expression patterns were detected based on daily GC dose. High dose GC (>18 mg/m²/day HCe; n=13), compared to physiologic (<12 mg/m²/day HCe; n=10) dose GC identified seven downregulated miRNAs: hsa-miR-320a, hsa-miR-122, hsa-let-7b, hsa-let-7i, hsa-miR-4747, hsa-miR-3591, hsa-miR-4732 (**Figure 1B**) (**Supplementary Table 1**). To characterize the expression of the differentially expressed miRNAs in all of the 37 patients who were receiving various GC doses, the miRNAs counts were normalized. A negative correlation (*P* ≤ 0.03) between GC dose and the expression of all seven miRNAs was found (**Figure 2**). These seven differentially expressed miRNAs were also evaluated according to GC dosecategory (**Supplementary Figure 1**). No significant differences in miRNAs expression were observed between high vs moderate GC dose and moderate vs physiologic GC dose.

### Enrichment analysis of differentially co-expressed miRNAs

**Figure 3:**
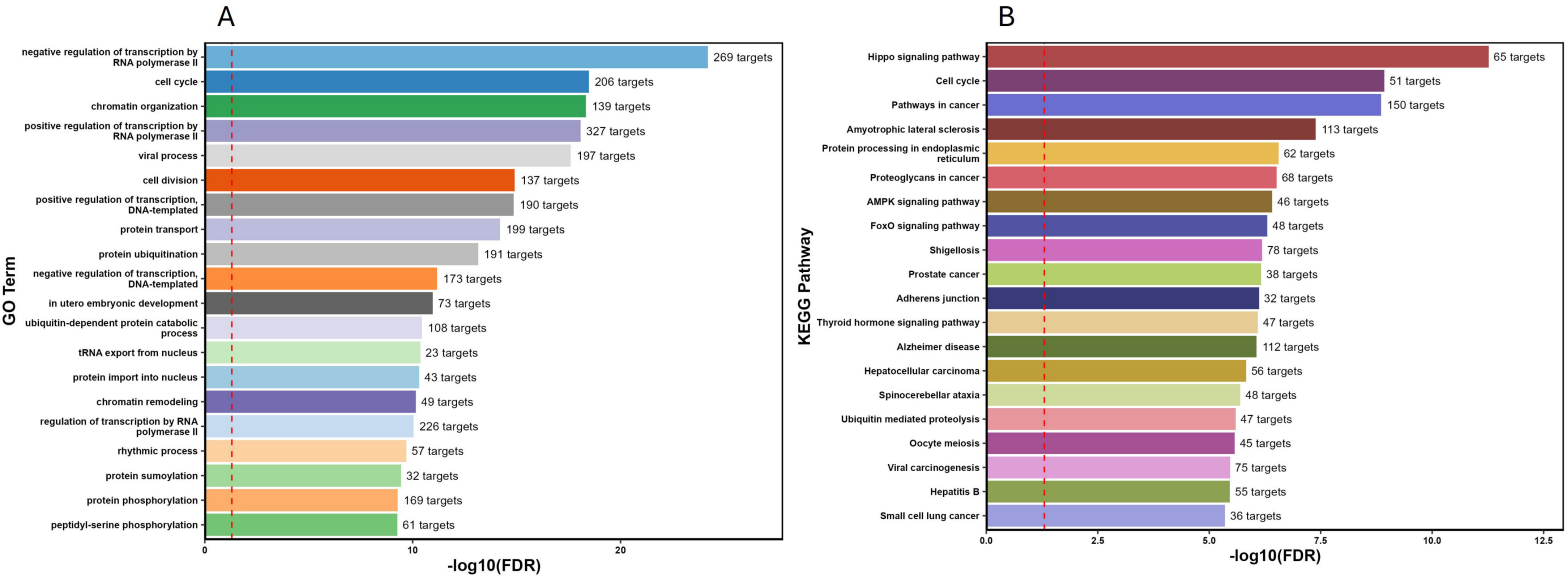
Functional enrichment analysis of differentially expressed miRNAs. **(A)** Gene Ontology (GO) enrichment analysis; bars represent biological processes (top 20 according to the adjusted *P-*value, respectively). **(B)** Kyoto Encyclopedia of Genes and Genomes (KEGG) Pathway enrichment analysis (top 20 most significant enrichment of the predicted target genes of the seven differentially expressed miRNAs).

Functional enrichment analysis of the seven co-expressed differentially expressed miRNAs revealed their biological roles by identifying the overrepresented pathways (KEGG), functions (GO terms), and disease associations of their predicted target genes. GO enrichment analysis showed differentially co-expressed miRNAs involved in multiple biological functions including responses associated with regulation of transcription, cell cycle progression, protein transport, and circadian rhythmic process (**Figure 3A**). To further study the possible pathways directly affected by the differentially co-expressed miRNAs, target genes were classified by KEGG pathway enrichment analysis. The top five related signaling pathways with 50 or more corresponding enriched target genes were selected. These targets involved a variety of signaling pathways associated with Hippo signaling, cell cycle progression, AMPK and FoxO signaling, and pathways involved in cancer (**Figure 3B**). These pathways were comprised of target genes involved in insulin resistance, lipid and glucose metabolism, and stress.

### Network and module analysis of co-expressed differentially expressed miRNAs

**Figure 4:**
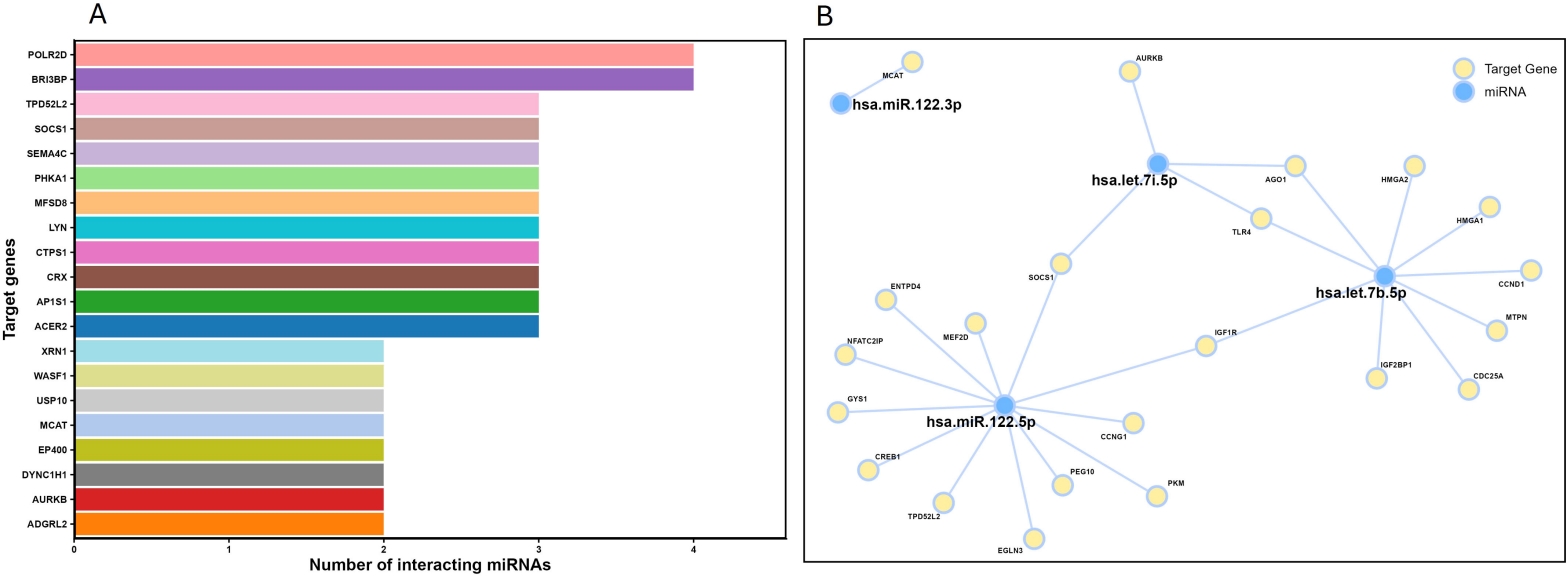
miRNA-target enrichment using Mienturnet analyses. **(A)** Bar plot showing the top 20 target genes identified and the number of interacting miRNAs. **(B)** Visualization of miRNA-target gene interaction network where blue circles refer to miRNAs, and yellow circles refer to target genes.

MIENTURNET identified 470 genes targeted by two or more differentially expressed miRNAs. Fifty-two of these genes were targeted by three differentially expressed miRNAs, and six of these genes were targeted by four differentially expressed miRNAs. The top 20 statistically significant target genes interacting with miRNAs were further evaluated (**Figures 4A, B**). The target pathway network analysis revealed that hsa-miR-122, hsa-let-7b, hsa-let-7i miRNAs were previously described in humans and shown to play a role in lipid metabolism and gluconeogenesis, with direct implications for insulin resistance and the development of metabolic syndrome. These miRNAs also play a role in oxidative stress, tumor metabolism, and cancer. Additionally, many target genes (*EGLN3, PKM, NFATC21P, CCNG1, TLR4, SOCS1, IGF1R, HMGA1, IGF2BP1, CDC25A, HMGA2, CCND1, AGO1, AMKB*) involved in immune response, cell cycle progression, cell proliferation, and cancer were targeted by these miRNAs (**Figure 4B**).

## Discussion

Exogenous GC treatment for adrenal insufficiency due to classic 21-OHD CAH is necessary, and the need for supraphysiologic dosing is common. However, chronic exposure to supraphysiologic GC can lead to many unwanted adverse outcomes (2). This is the first study to evaluate circulating miRNA profiles in CAH patients. MiRNAs regulate various biological processes. Knowledge about GC effects on miRNAs, their regulation, and their target genes is valuable in expanding our understanding of the impact of GC therapy on biological systems and in the development and evaluation of treatment strategies and novel biomarkers. In this pilot study, we identified differences between circulating miRNA profiles based on daily GC dose but not type of GC. Specifically, miRNA-320a, miRNA-122, miRNA-let-7b, miRNA-let-7i, miRNA-4747, miRNA-3591 and miRNA-4732 were negatively regulated by high GC dose, defined as >18 mg/m^2^/day HC equivalents. Utilizing enrichment analysis, we found that the differentially expressed miRNAs were mainly involved in cell cycle progression, metabolic dysfunction, circadian rhythms, and GC receptor (GR) signaling.

Previous studies have demonstrated that GCs can not only regulate the expression of miRNA but can also be modulated by miRNAs, especially influencing GR expression and its function, thus reducing GC cell sensitivity, increasing GC resistance, and targeting inflammatory pathways (35–38). Circulating miRNA profiles in patients are dynamic and have provided critical information about the development and progression of various diseases like cancer, and neurological and cardiovascular disease (14, 39–41). In our study, the miRNAs which were differentially expressed based on different GC regimens were previously shown to play a crucial role in inhibiting cancer cell proliferation, immune regulation, insulin resistance or metabolic syndrome, supporting the biological relevance of our findings (**Table 2**) (42–57).

**Table 2:** Differentially expressed miRNAs in various tissues.

miRNA	Identified tissues (42)	Associated conditions	Diseases where circulatory miRNA identified as a potential biomarker
hsa-miR-320a	Kidney, blood, adipose, heart, immune cells, liver, adrenal	Insulin resistance, cancer, angiogenesis	Metabolic syndrome (43) Cardiovascular disease (44, 45)
hsa-miR-122	Liver, adrenal, blood, adipose, immune cells, kidney, heart, arteries, pancreas	Metabolic syndrome, cell cycle progression, insulin resistance	Metabolic syndrome (46, 47) Inflammation/infection and sepsis (48–50)
hsa-let-7i	Adipose, arteries, heart, immune cells, liver, pancreas, blood, thyroid, kidney	Cardiovascular disease, cancer, neurodegenerative disorders, inflammation	Cerebral and cardiovascular diseases (51–53)
hsa-let-7b	Bladder, adrenal, digestive tract, liver, heart, arteries, uterus, fetal liver, lymph nodes, prostate, blood, immune cells	Vascular damage, ischemic injury, stroke, cancer	Cardiovascular conditions (54–56) Diabetes and insulin resistance (53)
hsa-miR-4747	Kidney, blood, adipose, ovary	Cell progression in cancer	
hsa-miR-3591	Liver, glioma cells, macrophages, exosomes	Glioma progression	
hsa-miR-4732	Blood, heart, lung, thyroid, liver, kidney	Lung cancer and other cancers	Cardiotoxicity (57)

In our study, some miRNAs which play an important role in cell cycle progression were differentially expressed with high doses of GC. The miRNA-320 family has been shown to be involved in modulating cancer cells by inhibiting cell proliferation and by promoting apoptosis (58) and is highly expressed in adrenal, liver, immune cells and blood. miRNA-320 plays a pivotal role in suppressing oncogenesis and has been suggested as a prognostic circulating biomarker in several cancers (58–60). Another key downregulated miRNA was miRNA-let-7b. This miRNA is well-established as a tumor suppressor by inhibiting cell proliferation, suppressing cell migration, and regulating cancer stem cells (61, 62). Studies have shown that miRNA-let-7i plays a vital role in various pathological conditions including cancer progression, cardiovascular disease, neurodegenerative disorders, and inflammatory conditions (61, 63). Both miRNA-3591 and miRNA-4732 have been shown to act as a tumor suppressors (64, 65).

MiRNA-320 has also been shown to mediate insulin resistance in adipocytes (66), play a role in the development of angiogenesis in diabetes (67), and act as a modulator of aquaporins in cerebral ischemia (68). In insulin resistant adipocytes, a 50-fold increase in miRNA-320 expression was found, and transfection with an antisense oligonucleotide against miRNA-320 resulted in increased insulin sensitivity (66). The miRNA profile identified in our study also included miRNA-122, a miRNA previously shown to play a role in metabolic syndrome (69) and GC action (70). MiRNA-122 was highly expressed in obese individuals and patients with metabolic syndrome and mediated the insulin resistance and abnormal glucose metabolism of human liver cells (69). In a cross-sectional study comparing obese and normal-weight children, miRNA-122 was identified as a candidate biomarker of childhood obesity (71, 72). In a randomized, crossover, single-blind study, 10 patients with primary adrenal insufficiency were studied during GC replacement and withdrawal, and miRNA-122-5p was identified as a possible biomarker of GC exposure (70). Furthermore, increased levels of miRNA-let-7b have shown to be associated with vascular damage (73), and miRNA-let-7i was shown to play a complex role in diabetes by regulating whole-body insulin sensitivity and acting as a potential prediabetes marker (74).

Based on our enrichment analysis, the downregulated miRNAs were not only involved in cell cycle progression, GC receptor signaling, and metabolic diseases, but also in rhythmic process including circadian regulation of gene expression. Previous studies demonstrated that miRNAs associated with clock genes, like *PER2*, promote oncogenesis in males by inhibiting the tumor suppressor pathways (75). Additional studies are needed to further understand the complex relation between miRNAs and circadian genes with exogenous GC administration. Studies of the interactions between GR signaling and circadian systems suggest that miRNAs target clock genes or GC signaling components to create a complex feedback loop where GCs influence the clock genes and miRNAs modulate the GC receptor activity, affecting rhythms in metabolism, immunity, development, and cancer (76–78). In our study, the downregulated miRNAs converged on signaling pathways critical for cell cycle progression and cell proliferation (**Figure 4**). For instance, miR-122-5p targets *CCNG*, *CREB1*, and its dysregulation may enhance the cell cycle progression.

Circulating miRNAs have been studied as potential biomarkers of adrenal diseases including Cushing’s Syndrome (CS) (14), adrenal tumors (79), primary bilateral macronodular adrenal hyperplasia (PBMAH) (80), and adrenal gland dysregulation during sepsis (81). Prior studies have shown that specific miRNA expression levels are altered in patients with CS in both circulation and pituitary tumors (14). Circulating miRNAs levels have been found to differ in patients with Cushing’s disease (CD) compared to those with CS due to ectopic ACTH secretion (82), in patients with CD compared to ACTH-independent CS (14), and in patients with hypercortisolism compared to patients with non-functioning adrenal tumors (83). Serum levels of miRNA have been shown to be a promising diagnostic tool to differentiate active adrenocortical carcinoma (ACC) and disease-free ACC (79). Our study was not designed to explore miRNA biomarkers specific to CAH. Rather, we explored GC effects on miRNA and identified a circulating miRNA signature associated with supraphysiologic GC dosing in patients with adrenal insufficiency due to 21-OHD CAH.

These findings represent the first description of a distinct circulating miRNA signature associated with supraphysiologic GC exposure in patients with classic CAH. The observed downregulation of key miRNAs, including those known to regulate insulin sensitivity (such as miRNA-122 and miRNA-320a) and cell-cycle progression (including members of the miRNA-let-7 family), suggests a plausible epigenetic mechanism that may contribute to the metabolic disturbances and potential long-term proliferative risks commonly seen with chronic high-dose GC therapy in CAH. If confirmed in independent cohorts, these miRNAs could emerge as valuable non-invasive biomarkers for detecting GC overtreatment, thereby facilitating more precise dose titration and helping to reduce cardiovascular, metabolic, and other treatment-related complications in this patient population.

Important limitations of our study include the small sample size and the inherent individual variability of a population of patients seen at one center which can impact miRNA expression. Variations in disease status and treatment history may have influenced the overall epigenetic effect causing changes in circulating miRNA levels. A key limitation was not evaluating a control population of individuals without CAH receiving GCs; this would help identify miRNAs affected solely by GC exposure. Moreover, synthetic GCs and hydrocortisone have variable biological actions and the grouping of all GCs together and the use of hydrocortisone dose equivalence may not have accurately captured the full effect of GC dose on miRNA expression. In addition, those receiving synthetic long-acting GCs were more likely to be receiving higher GC doses and, due to our small sample size, we were not able to fully analyze this potential confounder. We also did not have enough sample availability to perform qRTPCR validation. However, our goal was to perform molecular profiling of circulating miRNAs in CAH patients who are affected by their GC regimen and the use of hydrocortisone dose equivalents is used clinically (23) and in clinical trials (84, 85).

In summary, we report distinct miRNA expression profiles associated with supraphysiologic GC therapy compared to physiologic therapy in patients with adrenal insufficiency due to classic 21-OHD CAH. Our identified miRNAs target several mRNAs involved in insulin resistance, cell proliferation, and other biological processes. Identifying miRNAs and their targets associated with GC excess expands our understanding of the epigenetic mechanisms underlying adverse outcomes associated with supraphysiologic GC dosing in patients with CAH. Future work is needed to replicate our findings by including an independent validation cohort and identify the most clinically relevant miRNA biomarkers of GC excess.

## Data Availability

The miRNA-seq datasets generated for this study can be found in the National Center for Biotechnology Information BioProject (BioProject number PRJNA1442792), https://www.ncbi.nlm.nih.gov/bioproject/PRJNA1442792.

## Supplementary material

Supplementary Figure 1 Box-plot diagram showing normalized counts of miRNAs in patients on different glucocorticoid doses. A gradient relationship is observed, with miRNA counts decreasing across categories as glucocorticoid dose increases. The upper and lower limits of the boxes and lines inside the boxes indicate the 75^th^ and 25^th^ percentiles and median, respectively.
