# Supplementary material for: Exogenous glucocorticoid dose impacts circulating microRNA expression in patients with adrenal insufficiency due to 21-hydroxylase deficiency
Source: Front Endocrinol (Lausanne). 2026 Mar 31;17:1784619. doi: 10.3389/fendo.2026.1784619 (PMC13076110; doi:10.3389/fendo.2026.1784619)
Supplement: Supplementary file 1 [file Table1.docx]

**Supplementary Table 1: Significance Values of Differentially Expressed miRNAs**

| **miRNA** | **Log_2_(FC)** | ***P-*value** | **FDR** |
| --- | --- | --- | --- |
| hsa-miR-320a | -0.8948066 | 0.000020500 | 0.01041868 |
| hsa-miR-122 | -0.8116812 | 0.000131815 | 0.02400821 |
| hsa-let-7i | -0.8253403 | 0.000072900 | 0.02400821 |
| hsa-let-7b | -1.0371812 | 0.000018800 | 0.01041868 |
| hsa-miR-4747 | -0.8222228 | 0.000141641 | 0.02400821 |
| hsa-miR-3591 | -0.8116812 | 0.000131815 | 0.02400821 |
| hsa-miR-4732 | -0.6585636 | 0.000208896 | 0.03034957 |

FDR, false discovery rate; FC, fold change
