# Supplementary figures and images for: Exogenous glucocorticoid dose impacts circulating microRNA expression in patients with adrenal insufficiency due to 21-hydroxylase deficiency
Source: Front Endocrinol (Lausanne). 2026 Mar 31;17:1784619. doi: 10.3389/fendo.2026.1784619 (PMC13076110; doi:10.3389/fendo.2026.1784619)

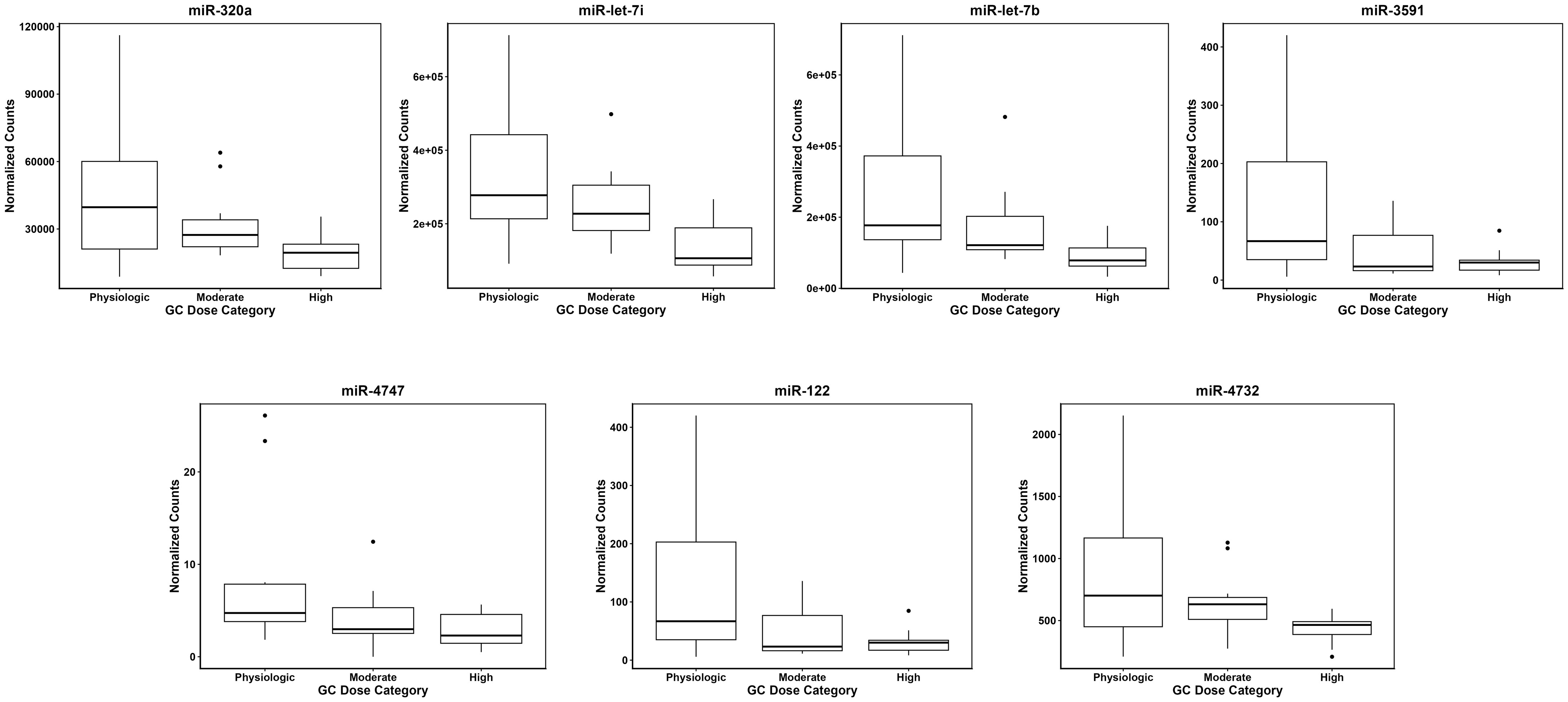

Supplement: Supplementary Figure 1 — Box-plot diagram showing normalized counts of miRNAs in patients on different glucocorticoid doses. A gradient relationship is observed, with miRNA counts decreasing across categories as glucocorticoid dose increases. The upper and lower limits of the boxes and lines inside the boxes indicate the 75th and 25th percentiles and median, respectively. [file Image1.jpeg]
